# Real-time and multimodality image-guided intelligent HIFU therapy for uterine fibroid

**DOI:** 10.7150/thno.42830

**Published:** 2020-03-26

**Authors:** Guochen Ning, Xinran Zhang, Qin Zhang, Zhibiao Wang, Hongen Liao

**Affiliations:** 1 Department of Biomedical Engineering, School of Medicine, Tsinghua University, Beijing, 100084, China; 2 National Engineering Research Center of Ultrasound Medicine, Chongqing, 401121, China.

**Keywords:** intelligent theranostics, HIFU therapy, multistage neural network, real-time lesion tracking

## Abstract

**Rationale**: High-intensity focused ultrasound (HIFU) therapy represents a noninvasive surgical approach to treat uterine fibroids. The operation of HIFU therapy relies on the information provided by medical images. In current HIFU therapy, all operations such as positioning of the lesion in magnetic resonance (MR) and ultrasound (US) images are manually performed by specifically trained doctors. Manual processing is an important limitation of the efficiency of HIFU therapy. In this paper, we aim to provide an automatic and accurate image guidance system, intelligent diagnosis, and treatment strategy for HIFU therapy by combining multimodality information.

**Methods**: In intelligent HIFU therapy, medical information and treatment strategy are automatically processed and generated by a real-time image guidance system. The system comprises a novel multistage deep convolutional neural network for preoperative diagnosis and a nonrigid US lesion tracking procedure for HIFU intraoperative image-assisted treatment. In the process of intelligent therapy, the treatment area is determined from the autogenerated lesion area. Based on the autodetected treatment area, the HIFU foci are distributed automatically according to the treatment strategy. Moreover, an image-based unexpected movement warning and other physiological monitoring are used during the intelligent treatment procedure for safety assurance.

**Results**: In the experiment, we integrated the intelligent treatment system on a commercial HIFU treatment device, and eight clinical experiments were performed. In the clinical validation, eight randomly selected clinical cases were used to verify the feasibility of the system. The results of the quantitative experiment indicated that our intelligent system met the HIFU clinical tracking accuracy and speed requirements. Moreover, the results of simulated repeated experiments confirmed that the autodistributed HIFU focus reached the level of intermediate clinical doctors. Operations performed by junior- or middle-level operators with the assistance of the proposed system can reach the level of operation performed by senior doctors. Various experiments prove that our proposed intelligent HIFU therapy process is feasible for treating common uterine fibroid cases.

**Conclusion**: We propose an intelligent HIFU therapy for uterine fibroid which integrates multiple medical information processing procedures. The experiment results demonstrated that the proposed procedures and methods can achieve monitored and automatic HIFU diagnosis and treatment. This research provides a possibility for intelligent and automatic noninvasive therapy for uterine fibroid.

## Introduction

Uterine leiomyomas (fibroids or myomas), the most common tumor in the female reproductive system, have a high prevalence (77%) and can cause reproductive dysfunction 1-3. Magnetic medical image-guided high-intensity focused ultrasound (HIFU) therapy has rapidly evolved in recent years as a noninvasive treatment for uterine fibroids that improves prognosis and avoids incisions. To achieve precision theranostics, multisource images should be processed intelligently to identify the lesion. However, diagnosis and operation in HIFU therapy currently largely depend on manual operation by specifically trained doctors, which represents a significant limitation for improving efficiency and accuracy. Automatic, accurate and real-time processing of magnetic resonance (MR) and ultrasound (US) images faces many challenges, especially in the clinical environment. For uterine fibroid diagnosis based on multisource 1T and 2T MR images, many obstacles remain in the accurate segmentation of MR images for HIFU treatment. In contrast to other kinds of tumors, uterine fibroids may vary in size and location. Additionally, the low contrast boundaries and image grayscale differences caused by the low quality of US images make detection of the lesion area difficult. Therefore, high-precision, automatic detection of uterine fibroids and intelligent HIFU focus distributing strategies are urgently needed.

Some studies of image-assisted surgery have focused on intraoperative image-guided therapy 4-5. Liao *et al.* proposed an integrated diagnosis and therapeutic system using intraoperative 5-aminolevulinic acid-induced fluorescence-guided robotic laser ablation for precision neurosurgery 6. For image-assisted surgery, many studies have combined different preoperative and intraoperative medical images to assist treatment, such as MR, computed tomography (CT) and X-ray images 7-8. These studies discuss some imaging methods and image utilization, but the processes of diagnosis and treatment have remained separate.

Noninvasive HIFU therapy has been widely studied for different lesion treatments. Seo* et al.* presented a visual tracking method for coagulation lesions in a moving target. They applied coagulation lesion tracking to a special US-guided robotic HIFU system, and motion compensation was investigated using a moving kidney phantom based on respiratory motion data. Their study focused on the use of HIFU systems for lesion identification and included model experiments 9. Although respiratory-induced organ motion compensation was added in follow-up work 10, and a kidney stone treatment experiment was carried out 11, the complications in the clinic were still not fully considered. Over the last few years, several studies and image processing methods for US video real-time processing have been reported 12-14. Pernot *et al.* used a triangulation method and three echoing signals 15. The results showed that a moving lesion can be located accurately by detecting the location of a single point. Oliveira* et al.* applied a US sensor to estimate the motion of a moving organ 16. However, sensor-based methods depend on special hardware systems and lack tumor location abilities. MR image segmentation is the main component of preoperative image-assisted diagnosis. Existing HIFU MR image segmentation methods are broadly classified into level set-based approaches 17, morphological operations-based approaches 18, and semiautomated-based approaches 19. For example, a semiautomatic-based approach based on a region- growing segmentation technique for uterine fibroid segmentation in MR-guided HIFU treatment was proposed 20 and tested in three cases. Rundo* et al*. used the above method for fibroid segmentation with a sensitivity of 84% 20. However, the methods proposed in these papers were not fully automatic; seed points needed to be manually selected but could not be selected accurately in some intractable cases. Fallahi *et al.* used the fuzzy c-means algorithm and morphological analyses to segment MR images of uterine fibroids in two steps and obtained an average Dice similarity coefficient (DSC) of 79.9% 21 in 10 cases. Although these methods achieved automatic uterine fibroid segmentation, the testing data reported in these papers were typical and limited.

In this research, we propose a multi-information fused automatic real-time image-guided diagnosis and treatment system for HIFU therapy. The system framework includes automatic uterine fibroid diagnostics; intraoperative US lesion tracking and intelligent HIFU focus distribution strategies. All image information involved in HIFU treatment is automatically processed and fused. The strategy of operation is generated based on the fused image information.

## Methods

### Intelligent HIFU treatment strategy

**Figure 1:**
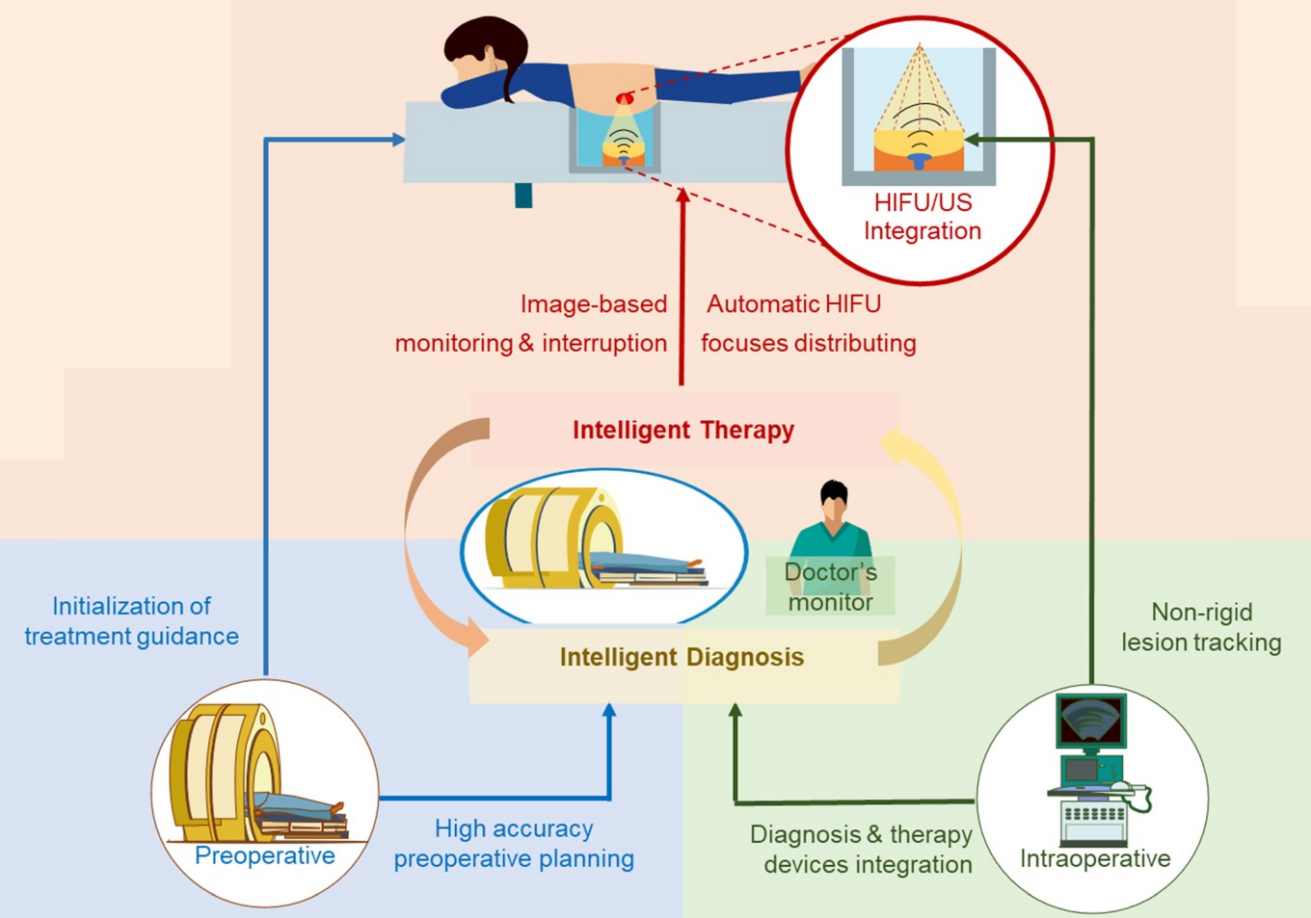
The proposed intelligent HIFU therapeutical framework is applied in the image-based HIFU diagnostic and treatment processing. The framework includes diagnosis and treatment parts. In the diagnosis part, morphology and spatial information in preoperative and intraoperative medical images can be accurately obtained and provided to the treatment strategy. In the treatment part, the HIFU focuses are distributed automatically by the proposed treatment strategy. For safety, the entire process is monitored by image-based monitoring step and doctors.

The proposed intelligent theranostics system is illustrated in Figure 1. As a kind of noninvasive therapy, medical images are the basic information for the operation. We aim to completely automate image processing and generate a reliable basis for diagnosis and treatment. Other factors, including physiological signals, reflect the current physical condition of the patient. The proposed image-guided HIFU therapy is implemented under the supervision and confirmation of a doctor.

**Figure 2:**
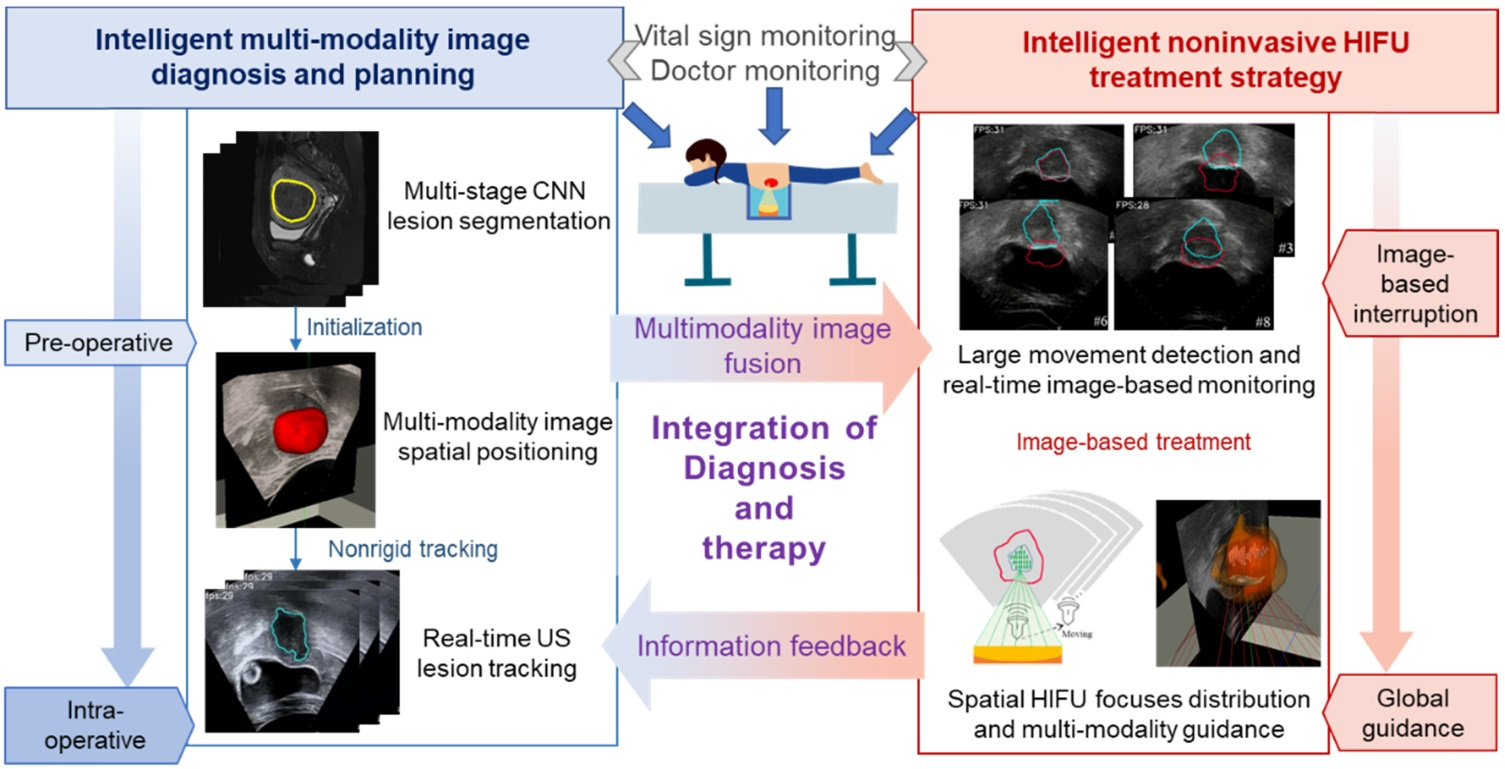
Intelligent HIFU treatment strategy for uterine fibroids. Diagnostic and planning information is generated from the automatic medical image processing system and provided to the HIFU treatment system. Automatic and manual monitoring is applied to ensure safety.

Our strategy for intelligent noninvasive HIFU therapy is shown in Figure. 2. Images information is extracted by the intelligent diagnostics and planning processing from the involved medical devices, including MR, US and HIFU devices. All this information is combined into a global information set and used to determine the treatment strategy. The HIFU probe treats the lesion area based on this information. The treatment strategy is monitored by medical images and physiological indicators. Additionally, the doctor further confirms and monitors all information during treatment to ensure safety.

To initialize the contour of the intraoperative lesion, accurate preoperative image segmentation at both the structural and sequence scales is needed. Intraoperative initialization requires both the structural information from the image and accurate spatial position correspondence. Therefore, we fuse and transfer the structure and position information from the preoperative image to the intraoperative stage. In the intraoperative stage, lesion information obtained from image processing is used as the primary treatment basis. The automatically identified lesion area is treated with the confirmation of the doctor. Warning and handling of accidents are also necessary. However, movement of the patient when she feels uncomfortable can lead to unexpected focus shifts, and the treatment process needs to be stopped when such events occur. Warnings about large movements are also sent. Finally, our intelligent HIFU therapy mode combines automatic image information processing confirmed by the doctor.

Joint preoperative and intraoperative information are combined with general lesion information for treatment. During the processing of medical images in HIFU therapy, the MR and US images are processed separately and combined with the results of the position information acquired from the HIFU device. The proposed procedure has four associated steps:

Step I: Preoperative diagnosis and planning. A novel multistage convolutional neural network is proposed to segment the uterine fibroids in the MR image for preoperative diagnosis. Three different parts of the network are connected to extract image features from the sequence scale, structure scale and pixel scale. Different stages of the network reduce the possibility of fibroid misjudgment by extracting effective lesion information.

Step II: Contour initialization of the intraoperative lesion region. The rigid registration is completed based on the spatial information of the HIFU device and the MR device. Then, the segmentation results obtained from preoperative MR images are used as the initial contour for subsequent tracking in the intraoperative US image.

Step III: Intraoperative lesion tracking. An improved morphological active contour without edges (MACWE) 22 method is used to deform the contour of the lesion in the real-time ultrasound scanning. To address the problem of poor processing of ultrasound images by the discrete and small morphological operators in the conventional method, we propose a special single operator.

Step IV: Based on the auto-outlined lesion area, the HIFU foci are automatically distributed in the autoidentified lesion area. An image-based auto unexpected movement warning and human monitoring are used to guarantee safety.

### Automatic preoperative image diagnosis and multimodal fusion

To achieve automatic preoperative image diagnosis, we propose a multistage convolutional neural network for MR image segmentation and a rigid MR-US registration procedure.

**Figure 3:**
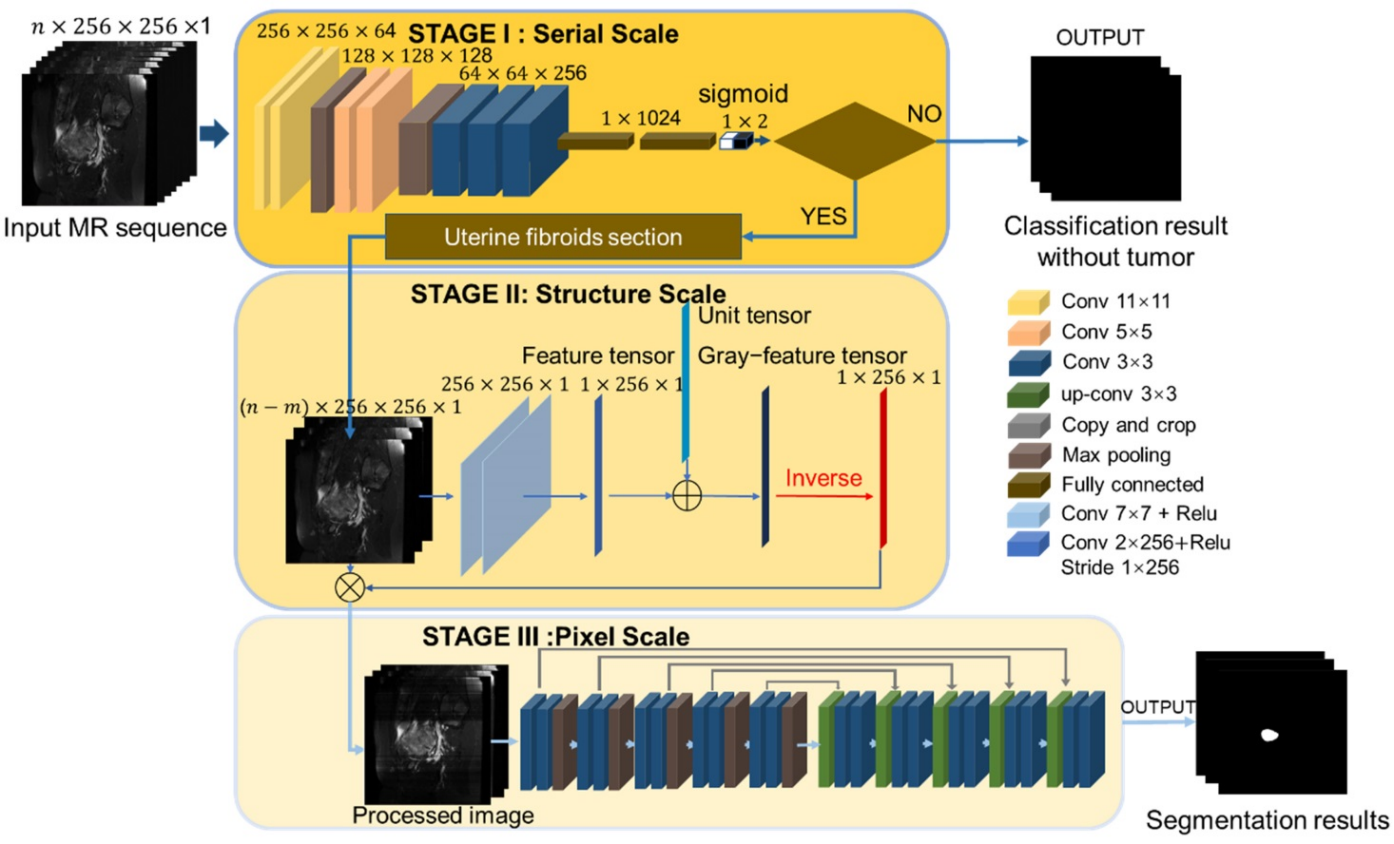
Multistage segmentation network. **Stage I**: Uterine fibroid classification task to obtain the fibroid section at the MR sequence scale. **Stage II**: Image structure processing task to reduce the influence from unrelated but remarkably similar regions in the image. **Stage III:** Multiscale feature segmentation task for uterine fibroid objects that contain the encoder path and decoder path.

The proposed segmentation network consists of three components as illustrated in Figure 3. The main problem of MR image segmentation is the locational uncertainty of uterine fibroids. To solve this problem, we use a pyramidal network structure to reduce the irrelevant parts at three image scales: the MR sequence scale, physiological structure scale and pixel scale. To perform these tasks, the neural network has three associated stages. Stage I: A classification network is harnessed to judge the existence of fibroids in the MR image and determine the section of the fibroids in the MR sequence, reducing the possibility of fibroid misjudgment in an integrated MR sequence. Stage II: A physiological structure processing convolutional network with a special structure automatically decreases the influence of unrelated but remarkably similar areas. Stage III: The processed image is input into the segmentation network. To improve the effectiveness of the processing network, we test two different baseline segmentation networks.

After the uterine fibroids are recognized and segmented from the MR image, the contour result is combined with the MR device's spatial information and the HIFU device's position information. The fused information, which consists of preoperative lesion contour and position information, is used to initialize the US video.

The initial contour of the intraoperative lesion tracking is obtained from preoperative MR segmentation results, spatial information of the HIFU device and intraoperative US video. The position of the patient during the operation is fixed. Thus, we can obtain the corresponding spatial information of the patient's body in the US image, defined as *P p U*, from the positioning system. Similarly, the position information of the human body in the preoperative MR image, *P p M*, can be obtained. The MR segmentation results can be set as the initial contour for the US lesion tracking relay on *P p U* and *P p M*. Thus, accurate MR segmentation is important.

#### Uterine fibroid section judgment network on the sequence scale

In the semantic segmentation task, the pixels are not equal in complexity, and the background or simple part accounts for most of the image 23. Because of the lower number of calculations, the accuracy and speed of target segmentation can be improved by distinguishing pixels in the image 24. Similar to the natural image, most of the images in the MR sequence have no lesions but have other structures. Segmenting all the images in the sequence not only increases the number of computations but also causes false positives in blank images. Therefore, we regard the image without uterine fibroids as the background of the MR sequence. A shallower classification network is proposed to dispose of the easier classification task and transfer the harder positions to a deeper segmentation network. The effective image information of the sequence scale can be extracted.

In classification tasks, an excessively deep classification network consumes more computer memory and results in degradation 25. A suitable convolutional neural network (CNN) model can analyze a hierarchy of feature representations. Different from natural image classification, the tumor judgment is a binary classification task, and the difference between the categories is also obvious. Therefore, an excessively deep network is unnecessary. Considering the complexity of tumor judgment and the high spatial resolution of the low-level features from shallow layers of the CNN, we designed a mathematical model of the CNN structure with lower complexity that is more suitable for uterine fibroid classification.

The model contains three convolution blocks and two fully connected blocks. Each convolution block consists of convolution, batch normalization (BN) and max-pooling layers. The sigmoid classifier, as the final output layer, produces the probability distribution of tumor judgment and maps the real number from 0 to 1. To train the classification network, we use an objective function derived from binary cross-entropy, which is suitable for binary classification with the use of the sigmoid. Because the ratio of the positive class and the negative class is imbalanced by 1:2, we add a weight to each class on the loss function. The loss function L_1_ is from the weighted binary cross-entropy for the classification network 26.

**Figure 4:**
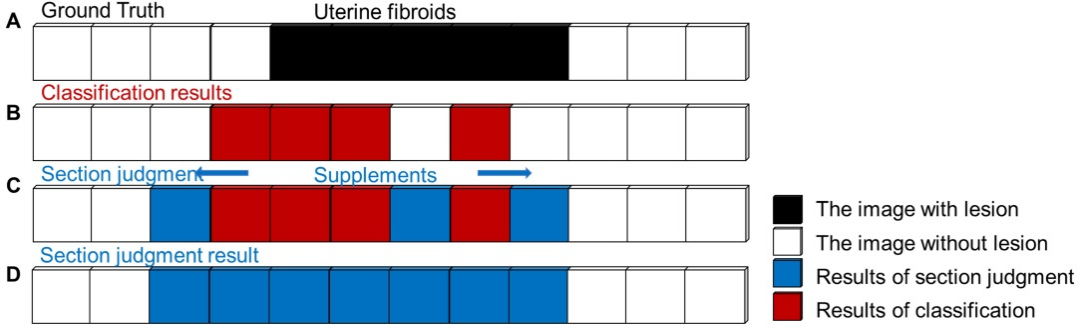
Schematic procedure of uterine fibroids section judgment. (**A**) Sample MR sequence with a true section (black). (**B**) Classification results in the MR sequence. (**C**) Adding supplements (blue) into the uterine fibroid section by a proposed procedure. (**D**) The final result of fibroid section judgment.

In clinical applications, false-negative errors of tumor judgment are inevitable and more severe than false-positive errors for diagnosis. To avoid the occurrence of false-negative errors in actual fibroid sections, the simple fibroid judgment is replaced by fibroid section judgment. The image before the first image with fibroids is defined as the beginning of the section, and the image after the last image with fibroids is defined at the end of the section. This procedure ensures that the true region of the lesion is covered as much as possible. The procedure of fibroid section judgment is shown in Figure 4.

#### Physiological structure and pixel scale uterine fibroid segmentation network

The HIFU clinical dataset used in the paper was provided by the National Engineering Research Center of Ultrasound Medicine. The MR dataset was collected from six different hospitals and five different MR scanners (uMR 570 1.5T scanner, UMI, China; MAGNETOM Verio 3.0T scanner, Siemens, Germany; MAGNETOM Sonata 1.5T scanner, Siemens; GE Signa HDXT 1.5T scanner, GE, USA; Philips Achieva 3.0T scanner, Philips, the Netherlands). The multisource causes variation in the image morphology. Occasionally, some regions that are extremely similar to uterine fibroids in grayscale and shape appear in the MR image. Such interference regions lead to misjudgment in the segmentation result. Conventional mathematical methods have some limitations for processing images with large morphological differences, such as a lack of generality. To solve the problems of data difference and image internal interference, we draw on the idea of domain adaptation and instance normalization 27-28 and propose a structural-scale processing network before performing the segmentation task.

The proposed physiological structure processing network aims to eliminate the disadvantages and reduce the adverse effects of image diversity, as illustrated in Figure 3. Building upon a hierarchical structure, the structure processing network contains two 7 × 7 convolution layers, which are not followed by the pooling layer as usual. Basic image features are extracted by a convolution kernel, and the level of the feature is positively related to the number of layers in the convolution. Different from the high-level extraction network, the role of the two convolution layers is similar to a low-pass Gaussian filter, and the convolution kernel is similar to a Gaussian kernel. The output feature map is the image with the details removed. The uterine fibroid MR image is shown in the sagittal direction, and the interference area more often appears in the longitudinal region.

As shown in Figure 3, the third convolution layer we propose is a linear convolution layer with a special convolution kernel with a size of 1×256 and a better receptive field on the *x* scale. Different from the previous two convolution layers, the third convolution layer extracts the grayscale value by the dot product value of the convolution kernel and the image matrix. Each value expresses the image grayscale feature on the *x* scale and constitutes the initial image in the gray feature tensor. To prevent calculation errors caused by a zero denominator in the dividing operation, we add a unit vector to the feature tensor and produce the gray feature tensor. The last step of the preprocessing network is to divide the image by the gray feature tensor and obtain the preprocessed image.

The architecture of the segmentation baseline consists of two parts, the encoder and decoder paths. Each path contains five blocks (two 3×3×3 convolutional, BN and max-pooling layers). To train the fibroid segmentation CNN model, we use the sigmoid classifier to semantically segment the image and determine the probability of whether the pixel belongs to the uterine fibroids.

The loss function we use is the DSC, which is optimized using the adaptive-moment-estimation (Adam) method 29. The Dice coefficient attaches more importance to the shared presence of the target area. The loss function L_2_ for the segmentation network is defined as


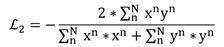
(1)

where the sums run over the N voxels of the predicted binary segmentation region y^n^∈Y and the ground truth binary region x^n^∈X. The DSC considers only the contribution of the shared target region of individual similarity and ignores the shared context. The DSC loss function has the advantage of minor calculations and is suitable for uterine fibroid segmentation 30.

The networks of stage II and stage III are trained together, and multiple stages are optimized together end-to-end. With forward propagation, the weight in both network parts can be adjusted simultaneously. The MR images in the experiments were resized to 256 × 256 in two dimensions. Each selected case included the following characteristics: 1) preoperative MR images of signal and multiple cases, 2) MR series collected by different MRI machines, 3) manual delineation of the fibroid contour by two experienced obstetricians on the source images. Finally, 320 cases were gathered with permission, and each case contained 25 images. To train the CNN model, 320 cases were randomly divided into three sets comprising 250 cases as the training dataset, 30 cases as the validation dataset, and 40 cases as the testing dataset. The model parameters were saved automatically when the highest validation accuracy was obtained. All CNN models were trained and tested on a workstation outfitted with an NVIDIA GeForce 1080 Ti Graphics processing Unit (GPU). We used an adaptive-moment estimation with a batch size of 4 and epochs of 200. During training, the learning rate was set as damped with an initial value of 0.01 and an attenuation rate of 0.9 in 1,000 global steps.

### Real-time HIFU ultrasound processing and focus distribution for uterine fibroid treatment

#### Nonrigid lesion tracking in intraoperative ultrasound video

**Figure 5:**
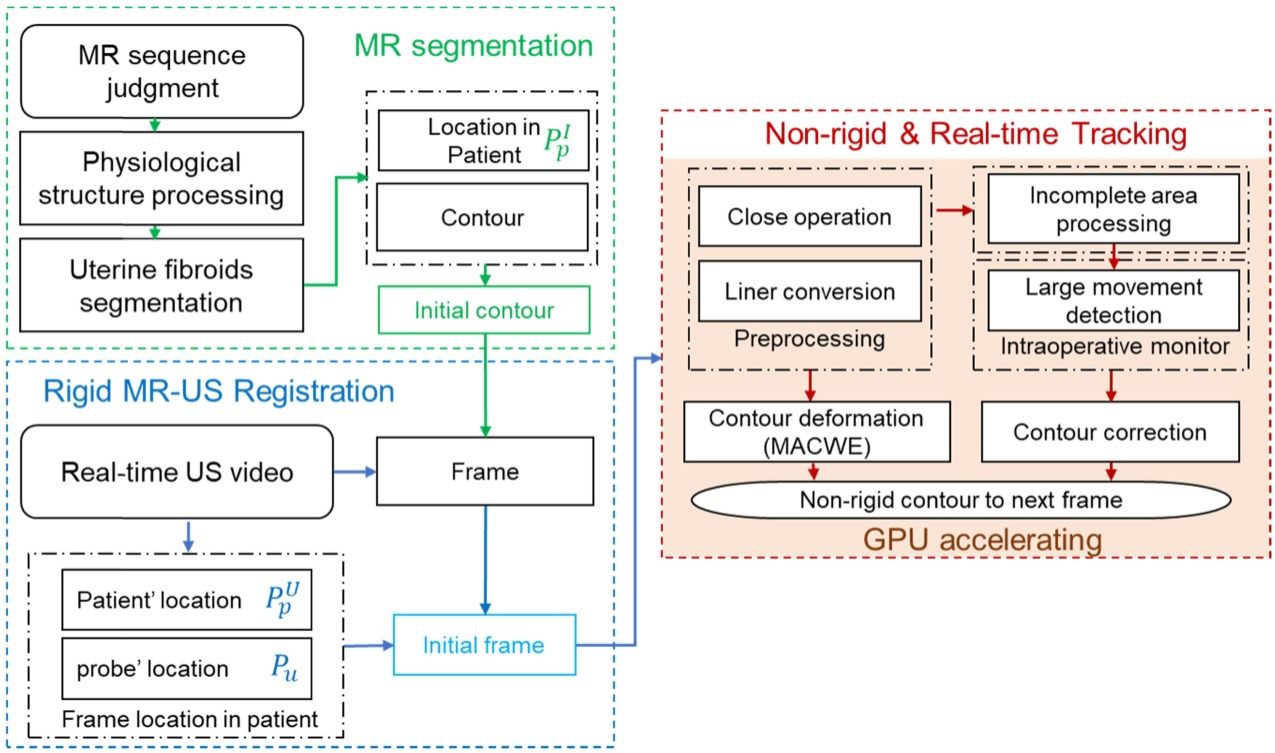
Global image guidance procedures for HIFU treatment. Rigid MR-US registration is based on the position information obtained from the HIFU device and preoperative MR image. Nonrigid and real-time US lesion tracking is under the compound detection of the lesion's contour and large movement. The entire framework is accelerated by GPU.

The demands of image-guided HIFU therapy are real-time processing and accurate lesion contour. The deformation of the contour is completed by MACWE. The entire framework of intraoperative US processing is illustrated in Figure 5.

Before the lesion tracking task, the original US image needs to be preprocessed. The focused ultrasound probe of the HIFU device is mounted in a sink that causes an echo in the US image. Unlike conventional ultrasound examination with the ultrasonic couplant, the HIFU ultrasound probe cannot be close to the patient's skin. The gap between the probe and the skin introduces noise in the ultrasound image. Thus, denoising and contrast enhancement of the image are required. In contrast to normal image noise, the echo speckle in the US image generally has a shape and a larger structure. We use a morphologically similar operation instead of ordinary image filtering.

An interfering structure that can easily be identified in HIFU ultrasound images is the bladder. The morphology of the bladder is similar to that of fibroids in US images due to the internal liquid. To improve image contrast and reduce disturbance, especially in the lesion, we use an efficient linear conversion to highlight the lesion and remove the bladder. The entire preprocessing function of an input US frame *In u* is


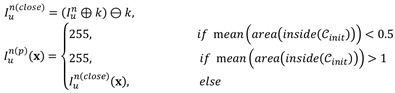
(2)

where κ is the kernel of similar operation and
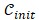
is the initial contour from MR segmentation results.

In many clinical cases, the lesion area in the intraoperative ultrasound is incomplete due to continuous tissue deformation, resulting in significant error that we call 'overflow' in the contours produced by the MACWE. To solve this problem, we added an incomplete area processing procedure in the level set generation step. The procedure detects the proportion of the level set. If most values are positive (inclined gradients) in the level set, the contours are essentially aligned with the real lesion area. The incomplete area processing is defined as


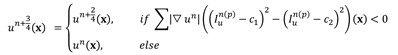
(3)

Based on the morphological active contours without edges (MACWE) model, we propose an improved method to specifically target the lesion area in HIFU ultrasound images. The basis points for the clinician to judge the lesion area in the ultrasound image are relative position, regional grayscale, and regional shape. Generally, the model-based image segmentation method requires accurate initialization of the contour, which is solved by the previous rigid MR-US registration. Thus, mainly the shape and grayscale characteristics of the lesion are considered.

In 22, morphological operators such as partial differential equations were shown to exhibit infinitesimal behavior, and a complex morphological operator was proposed to smooth the implicit hypersurfaces of the region. The preprocessed HIFU ultrasound image has a more pronounced contrast than the original image, but the speckles present in the image still have a large impact on the edge judgment. Therefore, we mainly consider the difference in grayscale inside and outside the contour. For a given curve *C* and image **I**, the function 31 can be represented as


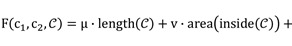

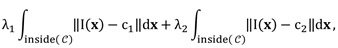
 (4)

where μ, v, λ_1_ and λ_2_ are the parameters of each term, and 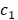
and 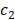 are the mean values inside and outside the contour of **I**


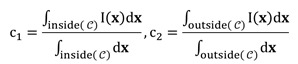
(5)

For a given preprocessed US frame *I n(p) u*, the current hypersurface in *I n(p) u* is defined as the level set 1/2 of a binary embedding function u^n^: Z → {0, 1} based on the initialized contour C n init The traditional single iterative morphological ACWE algorithm can be represented as


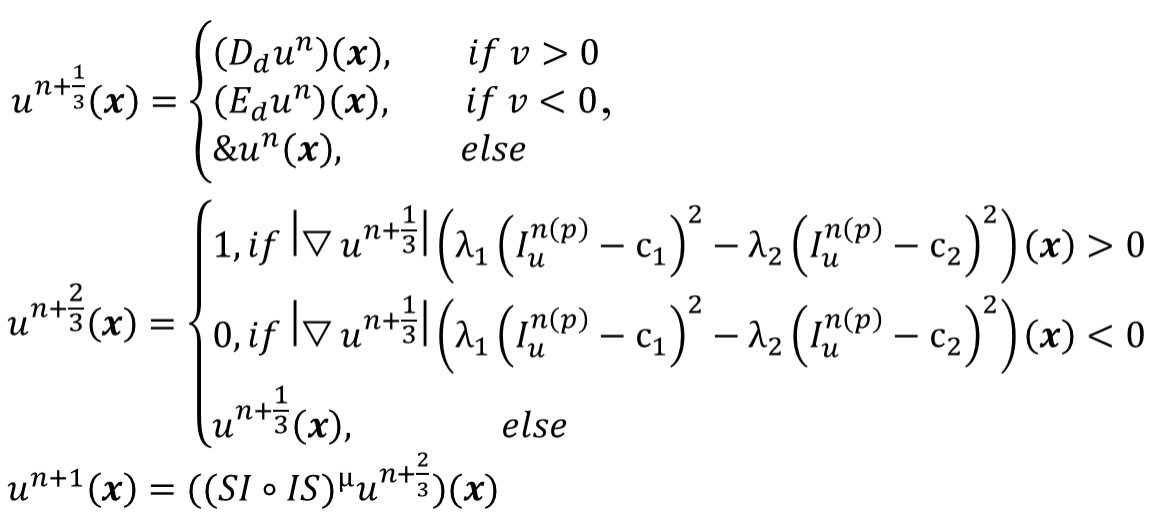
(6)

where *D_d_* and *E_d_* are the erosion and dilation operations, respectively. *SI◦IS* is the compound morphological operation.

To automate the process, we set image feature parameters μ, v, λ_1_ and λ_2_ as the unit values. Finally, the algorithm is represented as


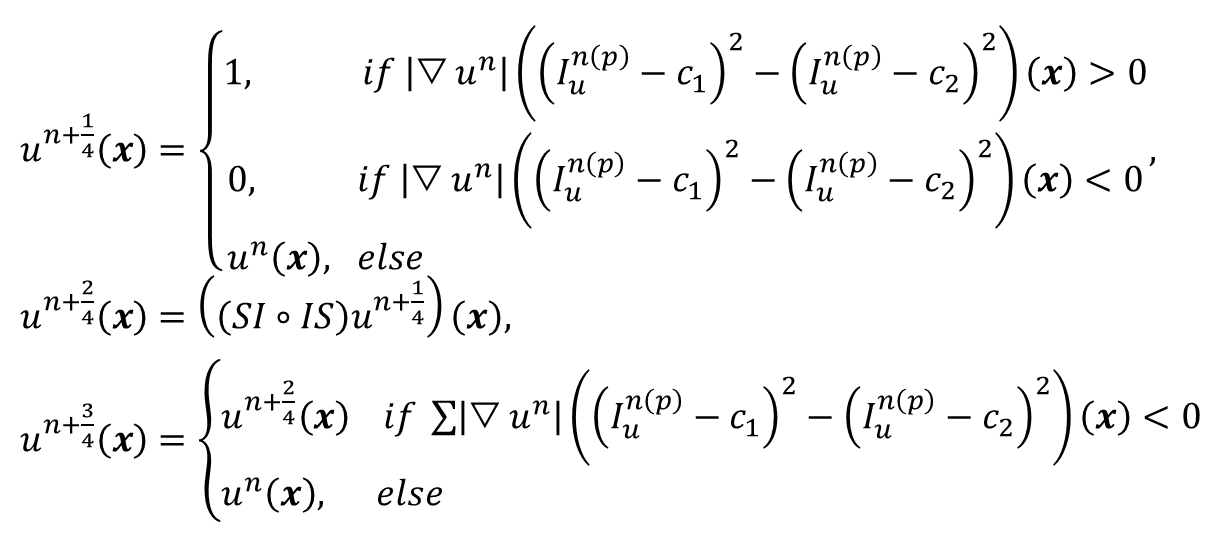
(7)

**Figure 6:**
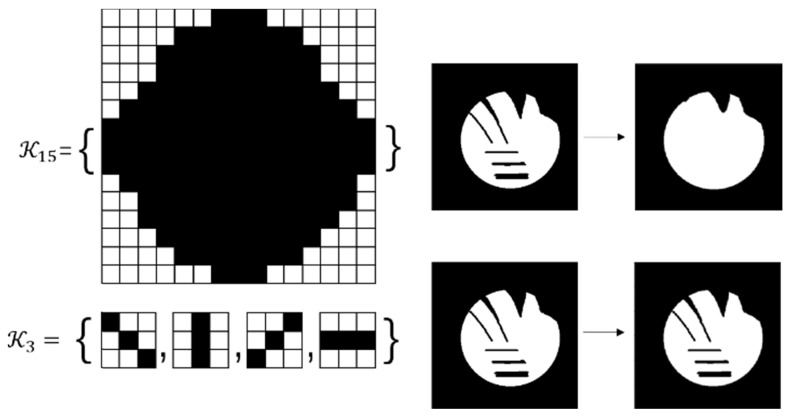
Comparison of the effects of the 𝒦_3_ and the 𝒦_15_ on internal structure processing. Compared with the conventional morphological operator 𝒦_3_, the proposed morphological operator 𝒦_15_ has a better effect on removing the irrelevant subtle structure due to its continuity and larger kernel.

In the previous algorithm, the morphological operations in *SI◦IS* consist of four discrete segments 𝒦_3_= {k 1 3, k2 3, k3 3, k4 3}. The operators are the length of three pixels and involve all shapes of the edge. This form of the operator has a good effect on natural image segmentation. The targets in the natural image usually have a clear relationship between structure and semantics. However, the uterine fibroids in the HIFU US image generally have internal structures. This kind of character causes grayscale differences inside the lesion. During the operation, the clinician mainly focuses on the outer contour of the uterine fibroids rather than the internal structure. Although existing discrete operators have better sensitivity to subtle structures, the small discrete operators obtain undesirable contours and make some mistakes, especially in HIFU ultrasound images with low signal noise ratio (SNR). To obtain the external contour of the lesion, we replace the 𝒦_3_ morphological operator by a single large segment 𝒦_15_= {k_15_} with a length of fifteen pixels. For larger-sized operators, smaller interference structures in the image can be removed during morphological operations. Additionally, the computational time of a single operator is less than four operators. The operator 𝒦_15_ and the effect of 𝒦_15_ on fine structure removal are shown in Figure 6.

The entire algorithm flow, including morphological operations, template matching, and mutual information, involves matrix operations that can be calculated by the GPU efficiently. We implement all matrix operations in the GPU by processing each pixel in the US image with one corresponding GPU thread. The parallel computing speed achieves triple or quadruple the improvement compared with central processing unit (CPU) processing and satisfies the real-time guidance requirement in the clinic.

#### Image-based HIFU intraoperative unexpected movement monitoring

In HIFU therapy, the patient is requested to lie prone on the HIFU device and avoid body movement. However, displacement during surgery is inevitable. Therefore, monitoring of large movement and timely interruption of treatment are necessary for intelligent HIFU treatment. To detect large movement in the current frame, mutual information (MI) detection is performed every 10 frames in the US video. For ultrasound video with 30 FPS, every-ten-frame detection implies a one-third second monitoring period to meet clinical monitoring requirements. The formula 32 for the current frame is


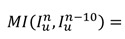



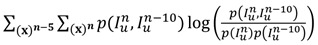
(8)

When a large movement is detected, the alarm message is sent and forces the treatment to stop until the image is stable. To maintain the tracking accuracy of lesions during large movement, we apply a template-matching method of normalized cross-correlation (NCC) 33 to find the best matching position with the initial contour in a limited range. Based on the empirical displacement of the lesion between frames, we can select the corresponding matching range [a, b]. The NCC results for image **I** and template **u** as random variables with samples *u_i_, l_i_, i* = a…b are defined as follows:


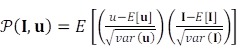
(9)

where *var*(**x**) and *E*(**x**) represent the empirical mean and variance for vectors **x**∈R^b-a^. After P is confirmed, the vector from P to the centroid of contour *C*^n^ is considered a moving force **m** with a parameter to α next frames.

Finally, the last step of tracking is defined as


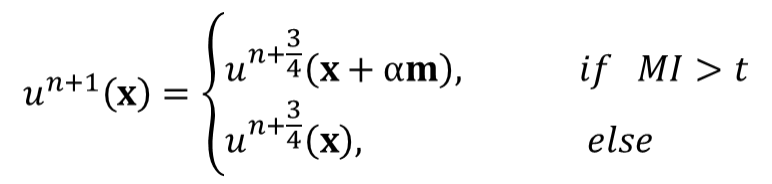
(10)

#### Image-based HIFU focuses on distributing for uterine fibroid treatment

In HIFU therapy, the focused ultrasound probe applies energy to the lesion area. To accurately automate the focus distribution based on the processed image, the ultrasound probe images the layered lesion area under the control of a controller. The ultrasound probes move at the same interval in the same direction, which spatially produces a layered ultrasound image and the entire abdomen. Finally, the 2D distribution in each layer is combined into a 3D focus distribution. Our method tracks the lesion area in the ultrasound image at the current location. Therefore, the proposed intelligent treatment plan is implemented separately in each layer of the ultrasound video.

**Figure 7:**
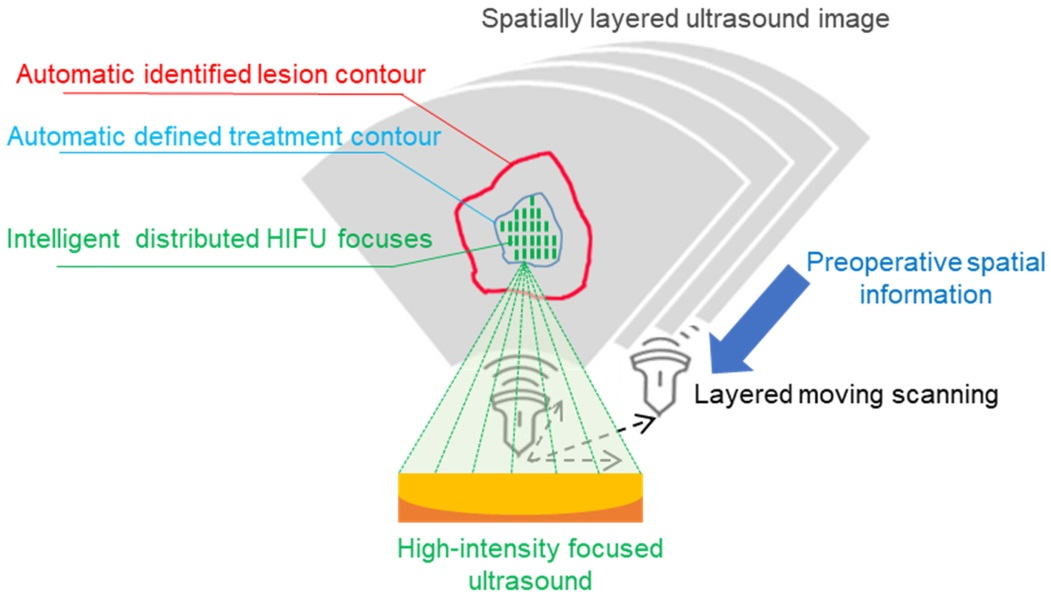
The proposed intelligent HIFU focuses on distributing the process. The spatially layered ultrasound images are obtained from a moving probe. We distribute the HIFU focus in the treatment area evenly and separately. The treatment area is defined as one-quarter of the center of the tracked lesion contour.

In the proposed intelligent HIFU treatment process, we deploy the focus distribution based on the real-time lesion contour. According to clinical experience, we choose one quarter of the center of the lesion contour as the intelligent treatment area. In this treatment area, the focus of the focused ultrasound is evenly distributed at 0.5 cm. The final focus is three-dimensionally distributed in the lesion based on the automatically identified lesion contour. All movements and focus distribution are intelligently completed based on fused information. The proposed image-based HIFU focus distributing strategy is shown in Figure 7.

## Experiments and Results

The experiment includes three parts: preoperative MR segmentation accuracy evaluation, intraoperative US lesion area tracking accuracy evaluation, and systematic clinical experiments. For the evaluation of intraoperative ultrasound lesion tracking, we first evaluated the accuracy and processing time of the lesion tracking method. Afterward, we evaluated the improvements in our proposed methods and procedures. Finally, we integrated our system with the clinical system to provide image-guided information to doctors in 8 clinical HIFU surgeries. To verify the feasibility of the intelligent HIFU therapy, we additionally evaluated our image-guided system to help improve the HIFU surgical learning curve.

### Evaluation of preoperative diagnostic information

The preoperative diagnostic information includes preoperative MR image segmentation and MR-US rigid registration. Because the accuracy of MR-US rigid registration is based on the accuracy of the HIFU and MR devices, we mainly evaluated the performance of the multistage MR segmentation network.

We present a series of experiments to analyze the impact of each of the main contributions and to justify the choices made in designing the proposed end-to-end segmentation network of uterine fibroids and the US tracking procedures. To quantitatively evaluate the performance of the present method, the DCS and Jaccard index (JI) 34 were employed as evaluation metrics.

We calculated the average values for DCS and JI in the testing dataset with the baseline (segNet) and the proposed structure processing network (Str- segNet) and entire network (Seq-Str-segNet) to validate the improvement of the structure scale processing stage and sequence scale processing. The quantitative comparison of the evaluation metric for each method is reported in Table 1. As shown in Table 1, the segmentation network with the proposed structure processing network achieved a better DSC and JI of 81.17±15.75 and 73.17±16.44. The large variations in the data source are reflected in a standard deviation (SD) of 15%-17%, but our method achieved a better SD of 1-2% than the method of comparison. The results from Table 1 indicate that the classification stage improved the DCS accuracy in the segmentation task by 7.41%.

**Figure 8:**
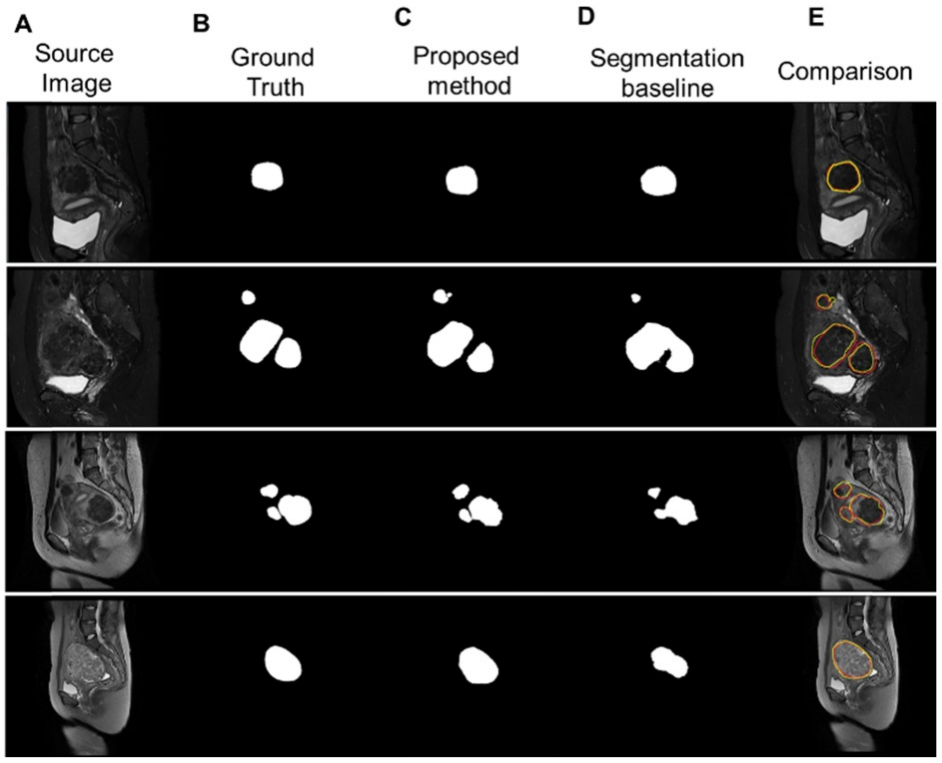
(**A**) Four sample MR images with different sources, including single and multiple. (b) The ground truth of the uterine fibroids. (**C**) The segmentation results of uterine fibroids obtained by the multistage segmentation network. (**E**) The segmentation results of uterine fibroids obtained by the baseline network. (**F**) Comparison of the ground truth (red) and segmentation results by the proposed method (yellow).

Figure 8 illustrates the segmentation results for four different types of uterine fibroids processed by our proposed multistage network and the segmentation baseline. Our method accurately delineated uterine fibroids in single, multiple and different types of cases with better results than the segmentation baseline.

### Performance of the real-time US lesion tracking

In the real-time US lesion tracking experiment, the accuracy of real-time lesion tracking procedures and the calculating efficiency for clinical HIFU guidance were evaluated. The computing platform included a CPU (Intel (R) Core (TM) i7-4790K) and a GPU (NVIDIA GeForce 1080 Ti). The corresponding US videos with HIFU device information were provided by the National Engineering Research Center of Ultrasound Medicine (JC200/300 Haifu treatment system, Haifu, China). We randomly selected 10 typical and clinical cases as the experimental US image data.

**Figure 9:**
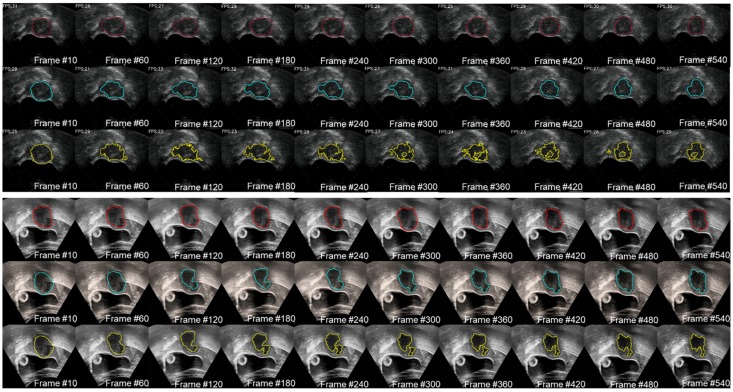
The gold standard of the two 20 s examples was qualitatively compared with the lesion tracking results of 𝒦_3_ and 𝒦_15_. The red contour is manually drawn, the yellow contour is generated by 𝒦_3_ and the blue contour is generated by 𝒦_15_.

**Figure 10:**
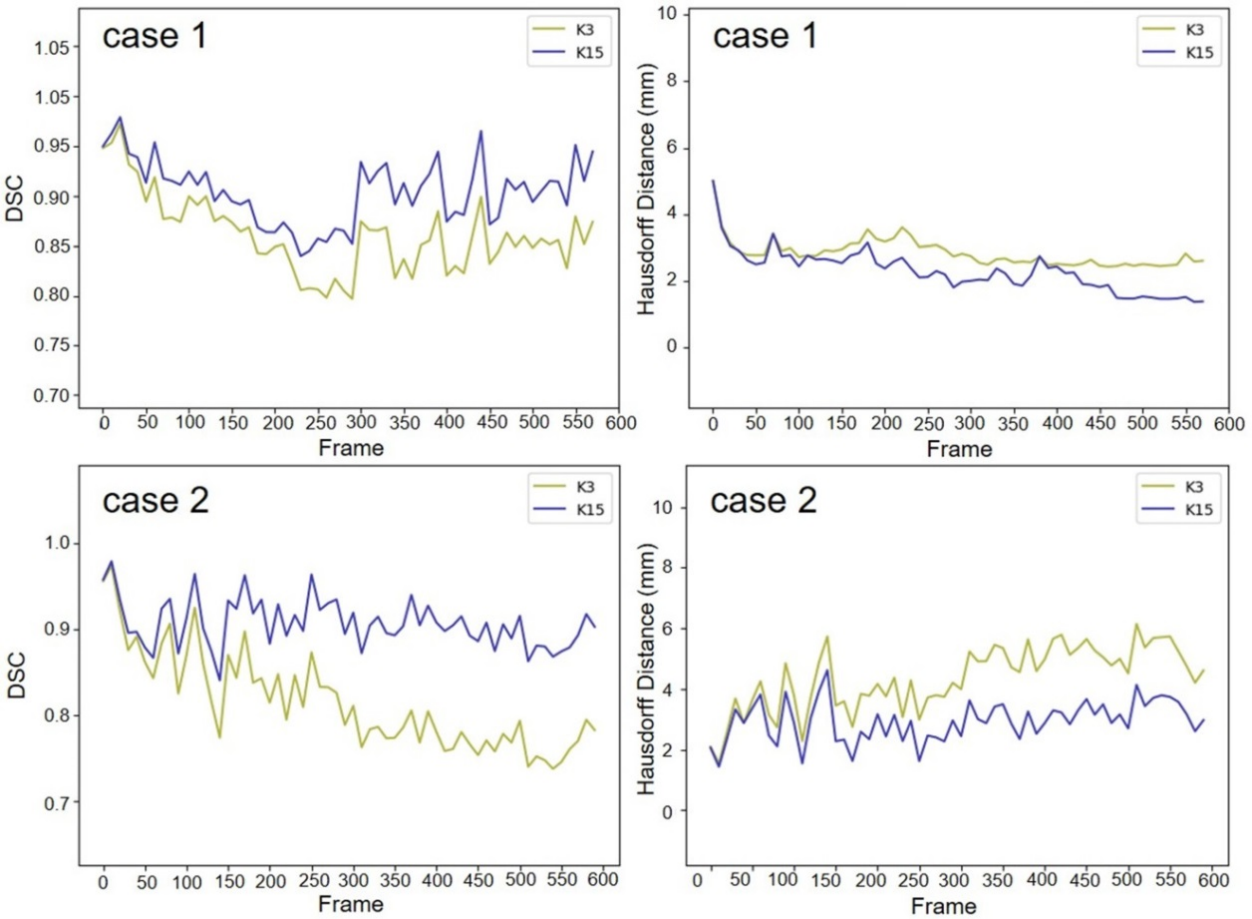
The gold standard of the two cases was quantitatively compared with the lesion tracking results of 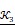 and 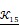. The result of 𝒦_15_ is better than 𝒦_3_ on DSC and HD.

We used two 20-second US videos manually labeled by the doctor (as the gold standard) to quantitatively evaluate the improved MACWE. The content was used to assess the accuracy of lesion tracking and the calculation efficiency. In the previous description, we proposed a single morphological operator 𝒦_15_ for MACWE instead of the traditional discrete morphological operator 𝒦_3_. To verify the rationality of the change, we compare the accuracy and efficiency of 𝒦_15_ and 𝒦_3_. The tracking results for two examples of 𝒦_15_ and 𝒦_3_ are shown in Figure 9. The first line is the gold standard, the second line is the tracking result of 𝒦_15_, and the third line is the tracking result of 𝒦_3_. The results show that the contour of 𝒦_15_ is more accurate than that of 𝒦_3_ and has better robustness. To quantitatively evaluate the results of two kernels, we used DSC, the Hausdorff distance (HD in mm) 35 and intersection over union (IoU) as the evaluation indicators. As shown in Figure 10 and Table 2, 𝒦_15_ provides better results comprehensively. In addition, we tested the computational efficiency of the two kernels on the same computing platform, as shown in Table 3. 𝒦_15_ is twice as fast as 𝒦_3_ on the GPU and more than three times faster on the CPU.

**Figure 11:**
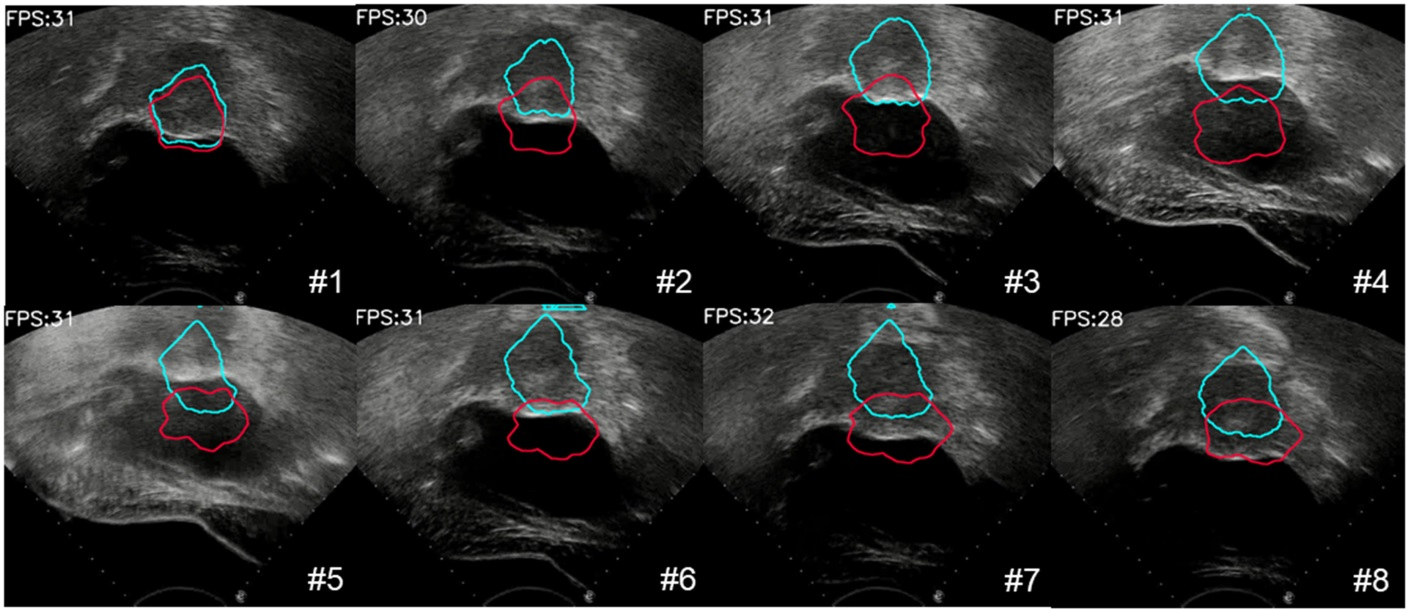
The process of large deformation detection to correct the contour of the lesion. The red contour was without large deformation detection, and the blue contour was with large deformation detection.

We also evaluated the proposed large displacement detection and incomplete area processing method. First, we verified the performance of large displacement detection as shown in Figure 11. The lesion area was displaced up and down due to the movement of the probe. A total of 8 frames were detected by the MI method as a large movement, and template matching was performed with the initial contour. The result, which was identified as valid, provided the moving force to the contour (blue contour) and had more accurate tracking results. By contrast, the contour without this procedure (red contour) completely missed the real lesion area. To visualize the results of lesion tracking, four different ultrasound lesion tracking videos are shown in Movie 1 (Supplementary Movie 1).

### Clinical experiments and evaluation of the feasibility of intelligent HIFU therapy

**Figure 12:**
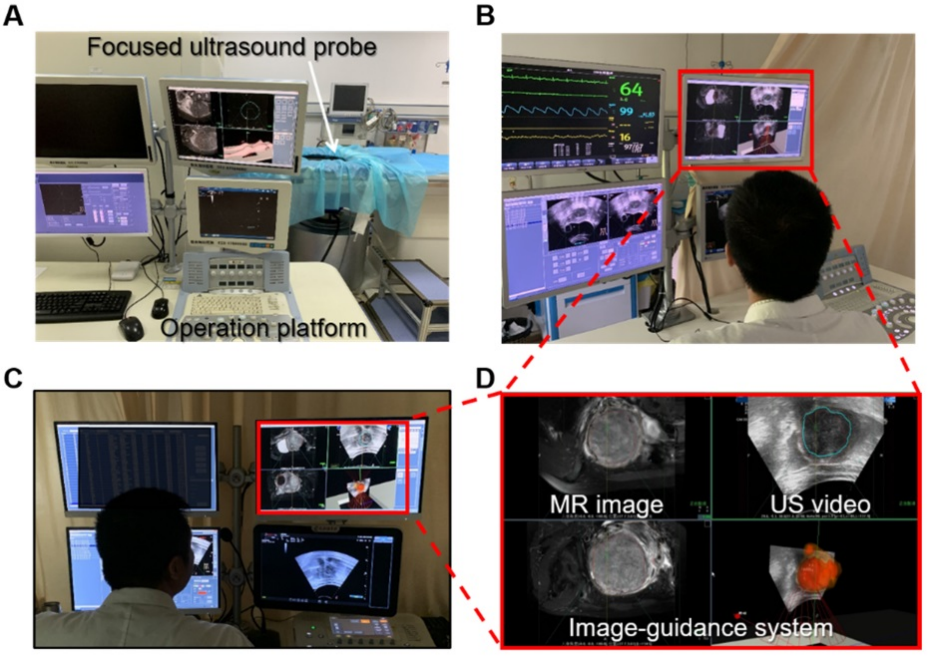
(**A**) is the system involved in the experiment, our image guidance system is integrated on the operating platform (**D**), and the patient is located on the device. (**B**) and (**C**) show the operation side of the surgical scene in the experiment. The patient is behind the isolation cloth.

To assess the feasibility of the proposed intelligent treatment strategy, we integrated the system with a commercial HIFU system (JC200/300 Haifu treatment system, Haifu, China) and performed clinical surgical validation. In the clinical trial, the operations were performed under the joint guidance and assistance of preoperative MR segmentation and intraoperative real-time ultrasound lesion contours. In the experiment, the HIFU focus was distributed according to the proposed intelligent treatment procedure. One doctor monitored and confirmed the position of the focus through the real-time US image. Additionally, another doctor simulated a traditional HIFU treatment procedure on the same patient's data, and the traditional HIFU focus distribution was used as the gold standard. The surgical side of the surgical scene is shown in Figure 12.

**Figure 13:**
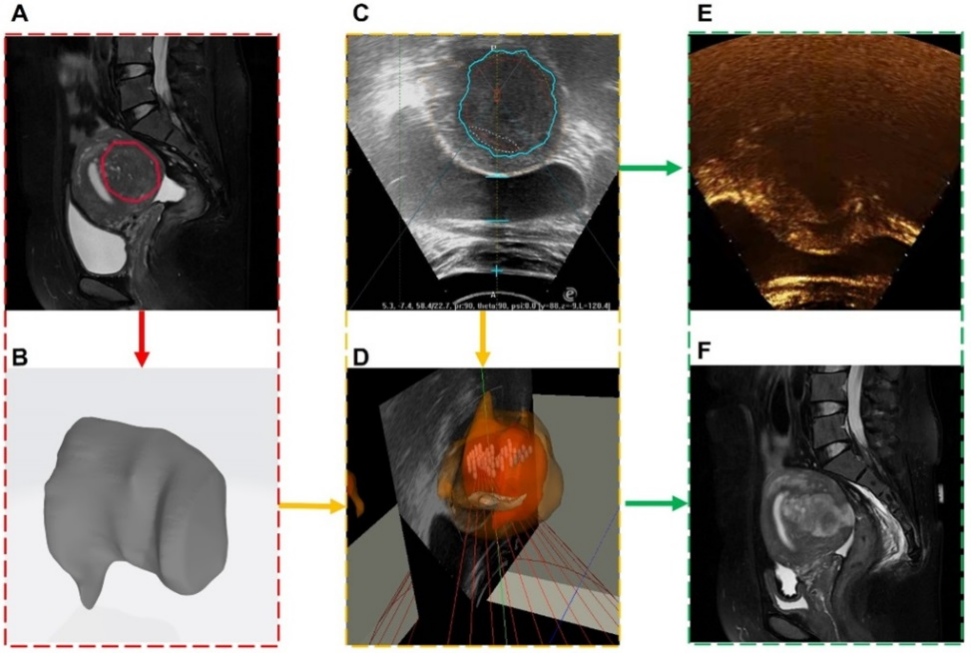
Medical image information contained in each clinical experiment case. Each case included (**A**) preoperative MR segmentation results and (**B**) 3D models, (**C**) intraoperative ultrasound images and (**D**) fused images, (**E**) postoperative ultrasound contrast, and (**F**) postoperative MR images.

A total of 8 clinical experiments were performed, and clinical information of the surgical procedure was recorded. Each case was randomly selected and included preoperative and intraoperative images, spatial coordinates and metrology of the intraoperative focused ultrasound focus, postoperative contrast-enhanced ultrasound, and MR images. An image-guided treatment process is shown in Movie 2 (Supplementary Movie 2). During the surgery, when the image guidance system showed that the focus (green point) was in the lesion area, the focused ultrasound probe applied energy (red point) at the focus. A medical image of one of the cases is shown in Figure 13. The red box is the preoperative MR segmentation result and 3D model, the yellow box is the intraoperative ultrasound video and image guidance system, and the green box is the postoperative contrast-enhanced ultrasound image and postoperative MR image.

**Figure 14:**
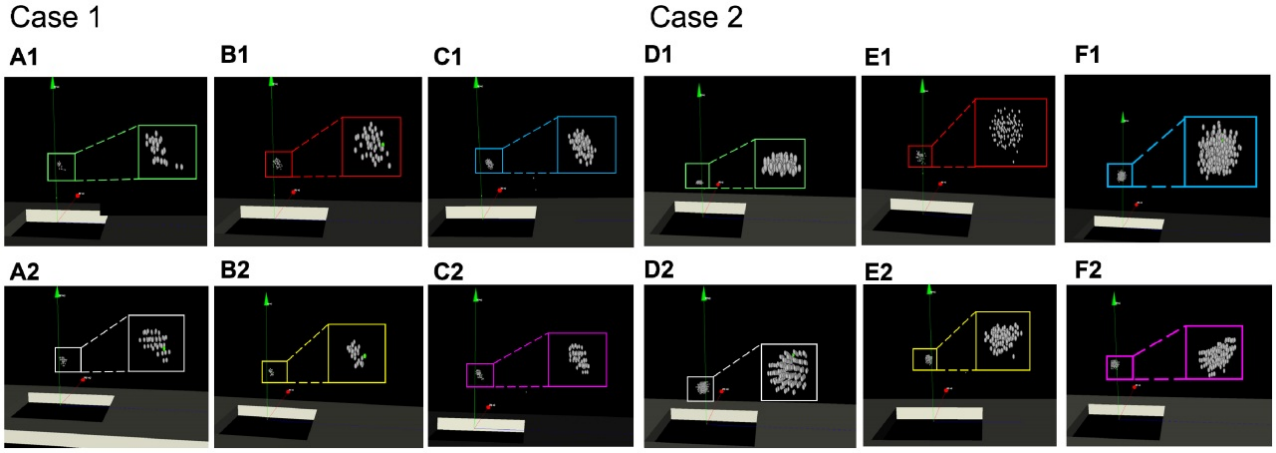
Comparisons of the spatial distribution of the HIFU focus that generated by different operators. Within the green box is the focus of the gold standard obtained by the senior doctor (**A**)(**D**). The white box is the focus of the intelligent treatment strategy. The blue box and the purple box are the simulated repeated focus of the junior operator. The red box and the yellow box are the simulated repeated focus of the intermediate operator. The upper results (**B1**)(**C1**)(**E1**)(**F1**) are generated without image guidance. The lower results (**B2**)(**C2**)(**E2**)(**F2**) are generated under image guidance.

Furthermore, we compared the focus distributions simulated generated by different operators. We used the focus performed by the senior doctor (Senior, with rich clinical experience) as the gold standard, including the distribution and number of foci. Foci automatically distributed by the proposed intelligent strategy were recorded (Auto). The procedure was then simulated by both an intermediate operator (Intermediate, with basic HIFU surgery training) and by a junior operator (Junior, without HIFU surgery training) with image guidance and without image guidance. Two examples of the experimental results are shown in Figure 14. The focus in the green box is the gold standard, and the focus in the white box is the intelligent distribution. The foci in the red and yellow boxes are the foci operated by the junior operator, and the purple and pink boxes are the foci of the intermediate operator.

As shown in Figure 14, the focus produced from the intelligent treatment strategy is even and closer to the intermediate-with-guidance's distribution. The intermediate-with-guidance distribution is closer to the gold standard in terms of quantity and spatial distribution. By contrast, the focus without image guidance is more dispersed, including beyond the region of treatment. To quantify the spatial similarity of the focus, the average value of the IoU in the focus of each layer of the US image was calculated. As shown in Table 4, there were fewer focal points under image guidance than without image-guided ultrasound. Additionally, the spatial position of the image- guided focus was more similar to the gold standard.

**Figure 15:**
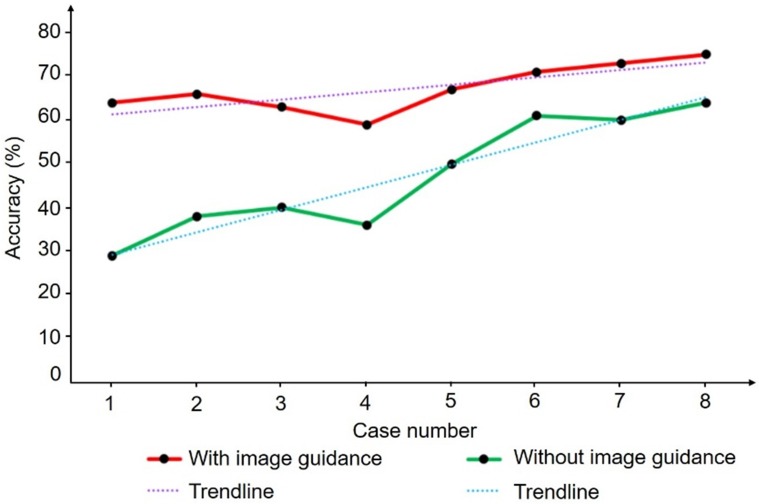
Two learning curves produced by two people with the same HIFU experience with or without image guidance.

We also evaluated the learning curves of two trainees with similar levels of understanding of HIFU and repeated eight cases in the same order with or without image guidance. Figure 15 shows the trend of focus accuracy for the two operators in the eight replicate experiments. The learning curve with image guidance has a slow upward trend and a high average similarity. By contrast, the learning curve without image guidance increases faster, but the similarity is generally lower. The trend of the learning curve indicates the rate at which the trainee obtains information from the learning process. The trainees obtained more accurate objective information and reduced their judgment when guided by images. Clinical experiments and repeated experiments showed that the proposed HIFU image guidance system can meet clinical needs and enable guidance and assistance of the surgical operation in real HIFU surgery. Additional experiments showed that the image guidance system can compensate for the inexperience of the operator to a certain extent and improve the accuracy and efficiency of the operation. Accurate image information can also help reduce learning costs.

## Discussion

In HIFU therapy, automatic, efficient, and precise processing of different medical information is vital. In this paper, we propose an intelligent and real-time HIFU theranostics strategy for uterine fibroids and applied this strategy in clinical treatment. Different medical images were processed and integrated by several novel methods and procedures. The treatment strategy generated valid HIFU focus distributions based on auto-obtained joint medical information. For preoperative diagnosis, we propose a novel multistage neural network for MR image segmentation. Different scale information of the MR image is screened by the network. The segmentation results of the MR images are transmitted to the treatment processes as the diagnostic information. In the intraoperative part, we integrate the spatial positioning information of the HIFU device with real-time processing of intraoperative ultrasound to provide image guidance for the doctor. In addition, we propose an improved MACWE method and multiple procedures to address clinical image problems. An image-based unexpected movement warning and other physiological monitoring options are used during the intelligent treatment procedure for safety assurance. Finally, the treatment strategy is generated from image-based intraoperative information.

To evaluate the efficiency of our image guidance system, we evaluated preoperative MR image segmentation accuracy and intraoperative ultrasound lesion tracking accuracy and performed clinical trials. The experiments confirmed that the entire pipeline could address complex data from clinical HIFU environments. In the evaluation of US lesion tracking methods, we compared the automatically obtained lesion contour with the manually labeled contour and showed that the proposed method obtained an accuracy of 90.67%. The evaluation experiments showed that the proposed framework not only had better robustness for HIFU ultrasound images but also had a faster calculation speed than the conventional method. The entire image calculating speed could reach 30 FPS. In clinical experiments, we integrated the image guidance system with the commercial HIFU system and performed 8 procedures. Clinical experiments on volunteers verified that the intelligent treatment strategy could achieve the basic distribution of HIFU foci. Moreover, the image guidance system effectively reduced the difficulty of HIFU operation based on evaluations of the learning curve.

The proposed intelligent HIFU theranostics strategy proved feasible in quantitative and clinical trials. However, at the current stage, the doctor still needs to confirm and monitor the operation and communicate with patients to ensure efficacy and safety during intelligent HIFU operation. In future work, more physiological signals will be integrated into the system for more comprehensive information acquisition. Moreover, the dosage of high-intensity ultrasound will be considered in the intelligent treatment strategy. To develop future intelligent HIFU theranostics, we will further focus on automatic surgical planning and the combination of decision- making and information. More intraoperative information will be included in subsequent studies, especially intraoperative feedback.

## Conclusion

In this work, we introduced an intelligent HIFU theranostics system. The system includes auto lesion detection in preoperative MRI and intraoperative real-time US images, an intelligent HIFU focus distribution strategy image-based unexpected movement monitoring. Several new proposed methods are used to ensure the accuracy and efficiency of the automatic theranostics procedure. Quantitative and qualitative experiments show that our system can accurately obtain lesion information and provide intelligent therapy in clinical cases.

## Supplementary Material

Supplementary movie 1.

Supplementary movie 2.

## Figures and Tables

**Table 1:** Results with different segmentation method for the uterine fibroids segmentation

Segmentation model	DSC	JI
Seq-Str-segNet	81.17±15.75	73.17±16.44
Str-segNet	79.78 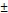 17.63	71.82 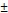 18.87
segNet	73.76 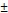 19.82	64.22 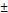 20.09

**Table 2:** The evaluation results of the proposed 𝒦_15_ and 𝒦_3_ in DSC, HD (mm) and IoU

Frame	𝒦ernel	𝒦_15_|𝒦_3_ DSC(case1)	𝒦_15_|𝒦_3_ HD(case1)	𝒦_15_|𝒦_3_ IoU(case1)	𝒦_15_|𝒦_3_ DSC(case2)	𝒦_15_|𝒦_3_ HD(case2)	𝒦_15_|𝒦_3_ IoU(case2)
#0		**0.95**|0.95	**1.40**|1.40	**0.93**|0.93	**0.95**|0.95	**1.40**|1.40	**0.93**|0.93
#100		**0.93**|0.92	**1.75**|3.50	**0.89**|0.91	**0.93**|0.92	**1.75**|3.50	**0.89**|0.91
#200		**0.92**|0.87	**2.45**|3.15	**0.88**|0.83	**0.92**|0.87	**2.45**|3.15	**0.88**|0.83
#300		**0.92**|0.78	**1.50**|3.15	**0.92**|0.80	**0.92**|0.78	**1.50**|3.15	**0.92**|0.80
#400		**0.90**|0.76	**1.75**|3.85	**0.89**|0.80	**0.90**|0.76	**1.75**|3.85	**0.89**|0.80
#500		**0.92**|0.71	**1.75**|5.25	**0.92**|0.81	**0.92**|0.71	**1.75**|5.25	**0.92**|0.81
#600		**0.93**|0.79	**1.75**|4.2	**0.88**|0.83	**0.93**|0.79	**1.75**|4.2	**0.88**|0.83

**Table 3:** Comparison of the computation speed of the proposed 𝒦_15_ and 𝒦_3_ in one iteration

Computing platform	𝒦_15_	𝒦_3_
CPU (ms)	20	68
GPU (ms)	8	16

**Table 4:** Comparison of the quantity and similarity of the HIFU focus distribution performed by the senior doctor, intermediate operation and junior operator

No.	Number of focuses					IoU (%)			
	Senior	Intermediate (w/ |w/o)	Junior (w/ |w/o)	Auto		Senior	Intermediate (w/ |w/o)	Junior (w/ |w/o)	Auto
1	25	50|63	33|44	48		/	61|57	59|36	63
2	96	106|125	98|113	103		/	65|55	53|37	67
3	53	76|89	63|77	71		/	71|59	55|42	73
4	32	41|54	41|49	39		/	73|62	47|33	75
